# Two new purplish species of Pezizales from the temperate forests of Mexico: *Marcelleina evangelinae* (Pezizaceae) and *Smardaea perezsilvae* (Pyropyxidaceae)

**DOI:** 10.3897/mycokeys.136.195741

**Published:** 2026-07-02

**Authors:** Tania Raymundo, Marcos Sánchez-Flores, César Ramiro Martínez González, Pedro Miguel Alvarez-Cortés, Jesús García-Jiménez, Martín Esqueda

**Affiliations:** 1 Instituto Politécnico Nacional, Escuela Nacional de Ciencias Biológicas, Laboratorio de Micología, Prolongación de Carpio y Plan de Ayala, Santo Tomás, Miguel Hidalgo, 11340, Cd. México, Mexico Centro de Investigación en Alimentación y Desarrollo A.C. Hermosillo Mexico; 2 Tecnológico Nacional de México, Instituto Tecnológico de Ciudad Victoria, Herbario Micológico José Castillo, Boulevard Emilio Portes Gil 1301, 87010, Cd. Victoria, Tamaulipas, Mexico Instituto Politécnico Nacional, Escuela Nacional de Ciencias Biológicas, Laboratorio de Micología Cd. México Mexico; 3 Centro de Investigación en Alimentación y Desarrollo A.C., Carretera Gustavo Astiazarán Rosas 46, La Victoria, 83304, Hermosillo, Sonora, Mexico Tecnológico Nacional de México, Instituto Tecnológico de Ciudad Victoria, Herbario Micológico José Castillo Cd. Victoria Mexico

**Keywords:** Histochemistry, mixed forest, phenolic derivatives, phylogeny, purple pigments

## Abstract

Two new species of Pezizales (Ascomycota) with violet-colored apothecia and inamyloid asci, growing on decaying wood and on soil, are described from Mexico. The first taxon, *Marcelleina evangelinae***sp. nov**., has reticulate and globose ascospores measuring 9–11 μm diam. It grows in mixed forests of the Sierra Madre Occidental. The second taxon, *Smardaea perezsilvae***sp. nov**., has equilateral to fusoid ascospores measuring 20–26 × 10–12 µm, crested with few pustules. It grows in the mixed forests of the Transversal Neovolcanic Axis. The histochemical data for both species indicate that their pigments contain phenolic groups. Descriptions, histochemistry, illustrations, phylogeny, and keys to both species are presented.

## Introduction

The genus *Marcelleina* Brumm., Korf & Rifai was erected by Brummelen (1967) in a monographic work on *Ascobolus* and *Saccobolus*, with *Marcelleina persoonii* (P. Crouan & H. Crouan) Brumm. as the type. Korf (1972) synonymized *Marcelleina* with *Pulparia* P. Karst., which was reinstated by Pfister (1985), while the revision of the type specimens by Dissing (1988) led to the synonymization of *Pulparia* with *Pulvinula* Boud. Initially, *Marcelleina* was considered in the family Pyronemataceae as a synonym of *Pulparia* (Korf 1972). Based on molecular data, it was placed in the family Pezizaceae (Hansen et al. 2001), confirmed by Hansen et al. (2005) and Van Vooren (2020).

Sammut et al. (2023) amended the genus description, characterized by epigeous, sessile, discoid, cupulate to pulvinate apothecia; violet to violet-brown, rarely depigmented to whitish; operculate, inamyloid, octospored asci with croziers; globose, hyaline, unigutulate ascospores, smooth or ornamented; and paraphyses with violet or brown internal pigments, sometimes with external pigment on the upper cell. Its terrestrial habitat is often on sandy or calcareous soils, both in coniferous (e.g., *Cupressus*, *Pinus*) and mixed forests, and often among humus and mosses.

Currently, seven species of *Marcelleina* are recorded (Index Fungorum 2025), most of which are described from Europe and one from Asia: *Marcelleina atroviolacea* Brumm. (Türkiye) (Uzun et al. 2018); *M. benkertii* J. Moravec (Czech Republic, Spain) (Moravec 1987); *Marcelleina brevicostatispora* J. Moravec (Moravec 1971); *M. chopraiana* (L.R. Batra) S.C. Kaushal (India) (Batra 1960); *M. donadinii* Astier & J. Moravec (France) (Moravec 1987); *M. georgii* (Svrček) J. Moravec (Czech Republic, Slovakia) (Moravec 1987); *M. mediterranea* Lantieri & Pfister (Italy, Spain) (Lantieri and Pfister 2010); *M. persoonii* (P. Crouan & H. Crouan) Brumm. (Czech Republic, France, Great Britain, Norway, United States of America) (Dennis 1981; Pfister 1985; Moravec 1987); *M. rickii* (Rehm) Gradon (Austria, Bulgaria, Czech Republic, France, Great Britain, Slovakia, Türkiye) (Uzun et al. 2018; Sammut et al. 2023); and *M. tuberculispora* K. Hansen & Sandal (Denmark) (Hansen et al. 1998). Likewise, Sammut et al. (2023) indicate that *M. mediterranea* is synonymous with *M. tuberculispora*. They found morphological similarities, and the LSU sequences of the *M. mediterranea* isotype and *M. tuberculispora* type show 99.16% similarity. In addition, *M. atroviolacea* is synonymized with *Smardaea planchonis* (Korf and Zhuang 1991). Van Vooren et al. (2026) proposed a new combination for *M. donadinii* as *Paradiscina donadinii* (Astier & J. Moravec) Martínez-Gil & Van Vooren.

The genus *Smardaea* Svrček was published by Svrček (1969), honoring the mycologist František Šmarda, with *S. amethystina* (W. Phillips) Svrček as the type. When Brummelen (1967) revised the type collection of *Ascobolus amethystinus* W. Phillips, he found two different species: *Ascobolus behnitziensis* Kirschst. and the fungus referred to *Peziza phillipsii* by Cooke (1876), but Svrček (1969) reinstated the original and priority epithet, *amethystina*, when he defined the genus *Smardaea*.

Zeng et al. (2022) established the family Pyropyxidaceae based on morphological and molecular data, segregating it from Pyronemataceae. The genus *Smardaea* was included in this new family. The genus is characterized by its discoid to cupuliform apothecia, colored with purple, blue-purple, violet, dark violet, black-purple to black hues; operculate, inamyloid, octospored asci, with uniseriate, globose or fusoid ascospores, with a smooth, verrucose, or tuberculate wall. Apothecia are found in sandy soil, leaf litter, mosses, and decaying wood (Zeng et al. 2022).

*Smardaea* consists of 11 species: *S. amethystina* from Great Britain (Brummelen 1969), *S. australis* P.S. Catches. & D.E.A. Catches from Australia (Catcheside et al. 2017), *S. isoldae* Raymundo & R. Valenz. from Mexico (Raymundo and Valenzuela 2021), *S. marchica* (Benkert & J. Moravec) Benkert from Germany (Benkert and Moravec 1986), *S. microspora* J.Z. Cao, L. Fan & B. Liu from China (Cao et al. 1990), *S. ovalispora* (Grelet) Van Vooren from France and Spain (Donadini 1976; Van Vooren 2009), *S. planchonis* (Dunal ex Boud.) Korf & W.Y. Zhuang from Argentina, Bermuda, Europe, and the United States of America (Seaver 1942; Hanlin et al. 1965; Donadini 1976), *S. protea* W.Y. Zhuang & Korf from the Czech Republic (Zhuang and Korf 1986; Benkert 2005), *S. purpurea* Dissing from Europe and China (Dissing 1985; Cao et al. 1990; Moyne et al. 2010), *S. reticulosperma* (Donadini, Riousset & G. Riousset) Benkert from France (Donadini 1986), and *S. verrucispora* (Donadini & Monier) Benkert from France and Italy (Donadini 1976; Agnello et al. 2019). The present study aims to describe two new species, *Marcelleina evangelinae* from the American continent and *Smardaea perezsilvae* from Mexico, using morphological, histochemical, and molecular data. Keys to accepted species of both genera are also provided.

## Materials and methods

### Specimens studied

The specimens were collected in 2021 in Yécora, Sonora, Sierra Madre Occidental, and in 2022 in Monte Tlaloc, Texcoco, Estado de México, Transversal Neovolcanic Axis. They are deposited in the herbarium of the fungal collection of the Escuela Nacional de Ciencias Biológicas, Instituto Politécnico Nacional (ENCB), and the José Castillo Tovar mycological herbarium of the Instituto Tecnológico de Ciudad Victoria (ITCV). Macroscopic features, such as the size and color of ascomata, were characterized. Microscopic observations of cross-sections of the middle part of the ascomata were analyzed and mounted on temporary slides. They were studied from dried specimens rehydrated in 70% alcohol, 5% KOH, and water. Microscopic characteristics were described according to Moravec (1987); both macroscopic and microscopic characters were recorded using descriptive statistics. The color of the apothecium structures was determined using the Methuen Color Manual (Kornerup and Wanscher 1978). New taxa were linked with the GBIF database (https://doi.org/gbif).

### Histochemical study

Cross-sections of the apothecia were placed in a 10% ferric chloride solution for 10 min to detect phenolic derivatives; the sample was then placed in distilled water and observed under light microscopy (LM) for gray-to-black-stained structures. To identify alkaloids, the sections were placed in Dragendorff’s reagent for 10 s, then washed in 10% hydrochloric acid for 30 s. The excess reagent was removed, and the sections were placed in 10% acetic acid and observed under LM for structures stained from deep orange to reddish-brown. Erlich’s reagent was applied to the sample to identify furan derivatives. A drop of concentrated sulfuric acid was immediately placed, and pink to orange spots were observed under LM. The sections were placed in a 4% ethanol-vanillin solution to detect terpenoids, then immediately treated with a drop of concentrated sulfuric acid. These were examined under LM, and structures stained red, magenta, violet, pink, orange, or blue were identified. Finally, for peptides, bromophenol blue was applied for 10 min, washed twice with distilled water, placed in 10% acetic acid, and observed under LM for blue-stained structures.

### DNA extraction, amplification, and sequencing

DNA was obtained from herbarium material. Genomic DNA was extracted using the Wizard Genomic DNA Purification Kit (Promega, Madison, WI, USA) according to the manufacturer’s protocol. The DNA was quantified with a NanoDrop 2000c (Thermo Scientific™, Wilmington, USA). Dilutions were prepared from each sample at 20 ng/µL to amplify the following five regions: the internal transcribed spacer rDNA-ITS1–5.8S rDNA–ITS2 (ITS), the nuclear large subunit ribosomal DNA (nLSU), the small mitochondrial subunit region (mtSSU), translation elongation factor 1-α (*tef1*), and beta-tubulin (*tub2*), using the primers shown in Table 1. The reaction mixture for PCR was prepared to a final volume of 15 µL containing 1× buffer, 0.8 mM dNTP mix, 20 pmol of each primer, 2 units of GoTaq DNA polymerase (Promega, USA), and 100 ng of template DNA.

**Table 1.** Primers used in this study.

**Loci/Segment**	**Primer**	**Sequence 5'-3'**	**T(°C)**	**Reference**
ITS	ITS5	GGAAGTAAAAGTCGTAACAAGG	58	White et al. 1990
	ITS4	TCCTCCGCTTATTGATATGC	58	
nLSU	LROR	ACCCGCTGAACTTAAGC	48	
	LR3	GGTCCGTGTTTCAAGAC	48	
*tub2*	βTubF	TCATTAGGTGGTGGAACGGG	60	Maitte et al. 2013
	βTubR	ATCACCATATCCTGGATCCC	60	
*tef1*	EF1-B-F1	ATYGCTTTAGAAAGTTYMTTTGC	53	Wu et al. 2014
	EF1-B-R	GGDATRAARWAWGARAARAARTG	53	
mtSSU	MS1	CAGCAGTCAAGAATATTAGTCAATG	63	White et al. 1990
	MS2	GCGGATTATCGAATTAAATAAC	53	

The PCR products were verified by agarose gel electrophoresis. The gels were run for 1 h at 95 V cm^–3^ in 1.5% agarose and 1× TAE buffer (Tris-acetate-EDTA). The gel was stained with GelRed (Biotium, USA), and the bands were visualized in an Infinity 3000 transilluminator (Vilber Lourmat, Eberhardzell, Germany). The amplified products were purified using the ExoSAP Purification Kit (Affymetrix, USA) according to the manufacturer’s instructions. They were quantified and prepared for the sequencing reaction using BigDye Terminator v.3.1 (Applied Biosystems, USA). These products were sequenced in both directions using an Applied Biosystems model 3730XL (Applied Biosystems, Foster City, USA) at the Instituto de Biología of the Universidad Nacional Autónoma de México (UNAM).

The sequences obtained were compared with the original chromatograms to detect and correct possible reading errors. The sequences of both strands of each gene were analyzed, edited, and assembled using BioEdit v.7.0.5 (Hall 1999) to generate a consensus sequence, which was compared with those deposited in GenBank (Arita et al. 2021) using the BLASTN v.2.2.9 tool (Zhang et al. 2000).

### Phylogenetic analysis

To explore the phylogenetic relationships of the new species of *Marcelleina*, an alignment was made based on the taxonomic sampling used by Sammut et al. (2023) (Table 2). Each gene region was independently aligned using the online version of MAFFT v.7 (Katoh et al. 2002, 2019; Katoh and Standley 2013). Alignments were reviewed in PhyDE v.10.0 (Müller et al. 2005), followed by minor manual adjustments to ensure character homology between taxa. The matrix was formed for ITS from nine taxa (690 characters), for nLSU from 16 taxa (831 characters), and for beta-tubulin (*tub2*) from seven taxa (370 characters). The aligned matrices were concatenated into a single matrix (17 taxa, 1,891 characters). Five partitioning schemes were established: one for ITS, one for nLSU, and three for the *tub2* gene region, all using the option to minimize stop codons in Mesquite v.4.02 (Maddison and Maddison 2025).

**Table 2.** GenBank accession numbers for sequences used in the phylogenetic analyses of *Marcelleina evangelinae* sp. nov. Accessions of the new species are indicated in bold.

**Species**	**Voucher**	**Country**	**ITS**	**nLSU**	***Tub*2**
*Ascobolus denudatus*	C: KS-94-146	Denmark	–	AY500528	AY513300
*Iodomarcelleina obscura*	LY: NV 2022.08.00	Malta	OR348398	OR348390	–
*Iodomarcelleina obscura*	C.S. 1091	Malta	OR348399	OR348391	–
*Ionopezia gerardi*	TL-5693	Denmark	–	AY500546	AY513332
*Ionopezia gerardi*	DHP-02.495	Mexico	–	AY500547	AY513333
Marcelleina cf. benkertii	L: 4343912	Netherlands	OR348393	OR348384	–
*Marcelleina benkertii*	CVL 070123-1	Spain	–	OR348385	–
***Marcelleina evangelinae***	**Holotype ENCB**	**Mexico**	**PZ53184**	**PZ531967**	**PZ53141**
*Marcelleina mediterranea*	K(M) 164532	Italy	OR348396	OR348388	–
*Marcelleina persoonii*	RM 2475	Spain	OR348394	OR348386	–
*Marcelleina persoonii*	KH 00.007 (C)	Denmark	–	AY500536	AY500463
*Marcelleina persoonii*	TL-5696 (C)	Denmark	–	AY500537	AY513311
*Marcelleina rickii*	LY:NV 2017.08.23	France	–	OL832154	–
*Marcelleina rickii*	LY:NV 2017.08.23	France	OM681379	–	–
*Marcelleina tuberculispora*	All-94-8 (C)	Denmark	DQ646535	AF335120	–
*Scotopezia pseudoanthracina*	KH 02.15 (C)	Norway	–	AY500538	AY513312
*Scotopezia pseudoanthracina*	LY:NV 2021.10.01	France	OR348397	OR348389	–

To explore the phylogenetic relationships of the new species of *Smardaea*, an alignment was made based on the taxonomic sampling employed by Zeng et al. (2022) (Table 3). Each gene region was independently aligned using the online version of MAFFT v.7 (Katoh et al. 2002, 2019; Katoh and Standley 2013). Alignments were reviewed in PhyDE v.10.0 (Müller et al. 2005), followed by minor manual adjustments to ensure character homology between taxa. The matrices were formed for nLSU from 12 taxa (811 characters), mtSSU from four taxa (640 characters), and translation elongation factor 1-α (*tef1*) from three taxa (670 characters). The aligned matrices were concatenated into a single matrix (12 taxa, 2,121 characters). Five partitioning schemes were established: one for nLSU, one for mtSSU, and three for the *tef1* gene region, all using the option to minimize stop codons in Mesquite v.4.02 (Maddison and Maddison 2025). Each region was independently aligned using the online version of MAFFT v.7.490 with the L-INS-i strategy for accurate alignment (Katoh et al. 2002, 2019; Katoh and Standley 2013). The alignments were reviewed in PhyDE v.10.0 (Müller et al. 2005), followed by minor manual adjustments to ensure character homology between taxa. The data were analyzed using maximum parsimony (MP), maximum likelihood (ML), and Bayesian inference (BI). Maximum parsimony analyses were carried out in PAUP* 4.0b10 (Swofford 2002) using the heuristic search mode, with 1,000 random starting replicates and TBR branch swapping, with MULTREES and Collapse on. Bootstrap values were estimated using 1,000 bootstrap replicates under the heuristic search mode, each with 100 random starting replicates.

**Table 3.** GenBank accession numbers for sequences used in the phylogenetic analyses of *Smardaea perezsilvae* sp. nov. Accessions of the new species are indicated in bold.

**Species**	**Voucher**	**Country**	**nLSU**	***tef-1*α**	**SSU**
*Jafnea fusicarpa*	420526MF0730	China	MH668008	–	–
*Jafnea fusicarpa*	HKAS90031	China	OP291096	–	OP291046
*Jafnea semitosta*	ISC 443551	USA	MT350430	–	–
*Micronematobotrys verrucosus*	O15-2161	China	FJ025221	–	FJ025224
*Micronematobotrys verrucosus*	E3-2181	China	FJ025222	–	FJ025225
*Planamyces parisiensis*	CBS 143165	France	MG386093	–	–
*Pyropyxis rubra*	K. Egger 289	Canada	DQ220404	KC109311	–
*Pyropyxis rubra*	K. Egger 323	Canada	DQ220405	KC109310	–
*Smardaea amethystina*	KH.97.132	Denmark	AF335176	–	–
***Smardaea perezsilvae***	**M. Sánchez 2980 ITCV**	**Mexico**	**PZ531968**	**PZ53181**	**PZ53102**
*Smardaea reticulosperma*	MPU: JDC 265-84	France	MT273649	–	–
*Smardaea verrucispora*	AMB 17161	Italy	MK025554	–	–

Maximum likelihood analyses were conducted in RAxML v.8.2.10 (Stamatakis 2014) using the GTR + G model of nucleotide substitution. To assess branch support, 1,000 nonparametric rapid bootstrap pseudoreplicates were generated using the GTRGAMMA model. For Bayesian posterior probability, the best evolutionary model for the alignment was sought using PartitionFinder (Lanfear et al. 2014, 2017; Frandsen et al. 2015). Phylogenetic analyses were performed using MrBayes v.3.2.6 (Huelsenbeck and Ronquist 2001; Nylander et al. 2004). The information block for the matrix included two simultaneous runs of Monte Carlo chains at a temperature of 0.2, sampling 10 million generations (standard deviation ≤ 0.1), and trees every 1,000 generations. Chain convergence was visualized in Tracer v.1.6 (Rambaut et al. 2014). The maximum credibility phylogram of the clades recovered with TreeAnnotator v.1.8 (Bouckaert et al. 2014) with a 25% burn-in was chosen. The phylogenetic trees were edited using FigTree v.1.4.3 (http://tree.bio.ed.ac.uk/sftware/figtree/).

## Results

### Phylogenetic analysis

*Marcelleina evangelinae* Raymundo, Sánchez-Flores, Alv.-Cortés & Esqueda, sp. nov.

The combined ITS+nLSU+*tub2* dataset comprises 17 taxa and 1,891 positions, including gaps. Three phylogenetic analyses, MP, ML, and BI, were conducted and resulted in generally congruent topologies. The best RAxML tree, with a final likelihood value of –19562.0631, is presented. The matrix contained 1,520 distinct alignment patterns, with 10.19% of characters undetermined or gaps. Estimated base frequencies were as follows: A = 0.056210, C = 0.168320, G = 0.234975, T = 0.206833; substitution rates AC = 1.190000, AG = 2.920467, AT = 1.103822, CG = 1.998620, CT = 6.960244, GT = 1.000000; gamma distribution shape parameter α = 0.198522. In the Bayesian analysis, the standard deviation between the chains stabilized at 0.008 after 3.9 million generations.

**Figure 1.**
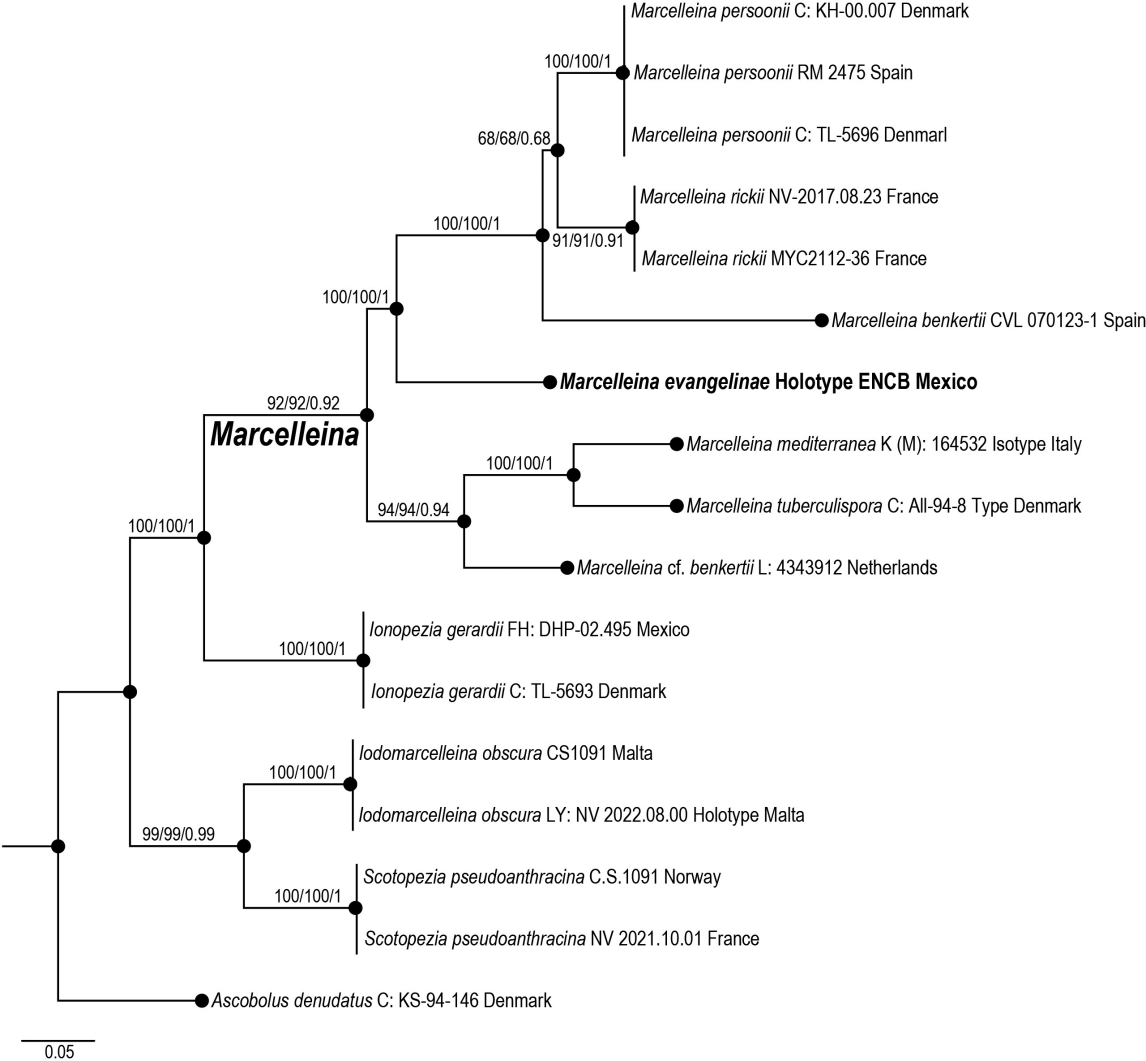
Phylogenetic reconstruction based on the concatenated ITS + nLSU + *tub2* sequence alignment of *Marcelleina evangelinae*. Maximum parsimony and Bayesian analyses recovered identical topologies concerning the relationships among the main clades of *Marcelleina* members. For each node, the following values are provided: maximum parsimony (MP ≥ 90%, left), maximum likelihood bootstrap (ML ≥ 90%, middle), and Bayesian inference posterior probability (BIPP ≥ 0.90, right). *Ascobolus denudatus* was used as the outgroup. The scale bar represents the expected number of nucleotide substitutions per site. The phylogenetic position of *Marcelleina evangelinae* is shown in bold.

No significant changes in tree topology, trace, or cumulative split frequencies of selected nodes were observed after approximately 4.5 million generations, so the first 2.5 million sampled trees (25%) were discarded as burn-in. To confirm that the sample size was sufficient, the parameter file was examined in Tracer v.1.7 (Rambaut et al. 2014), and it was confirmed that all parameters had an estimated sample size above 1,500. Maximum parsimony, maximum likelihood, and Bayesian inference recovered *M. evangelinae*, supporting the existence of one new taxon distinct from related species of *Marcelleina* (BS = 100%, BI*p* = 1) (Fig. 1), forming a highly supported monophyletic group.

#### *Smardaea perezsilvae* Sánchez-Flores, J. García, Esqueda & Raymundo, sp. nov.

The combined nLSU+mtSSU+*tef1* dataset comprises 12 taxa with 2,121 positions, including gaps. Three phylogenetic analyses, MP, ML, and BI, were conducted and resulted in generally congruent topologies. The best RAxML tree, with a final likelihood value of –11205.956342, is presented. The matrix contained 1,021 distinct alignment patterns, with 8.07% of characters undetermined or missing. Estimated base frequencies were as follows: A = 0.005622, C = 0.116824, G = 0.203821, and T = 0.29254; substitution rates AC = 1.920356, AG = 2.000281, AT = 1.830245, CG = 1.036024, CT = 6.053256, and GT = 1.059862; gamma distribution shape parameter α = 0.103540. In the Bayesian analysis, the standard deviation between the chains stabilized at 0.0003 after 2.1 million generations.

**Figure 2.**
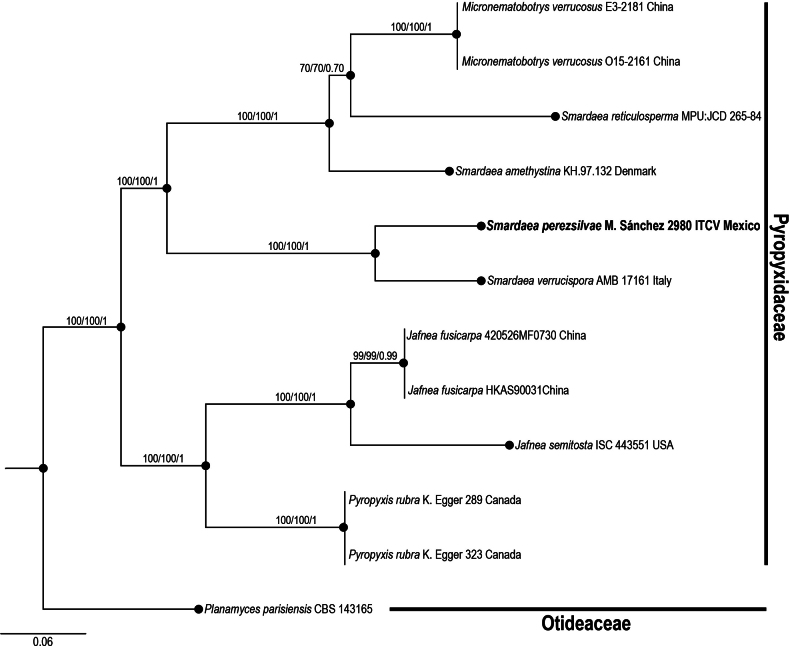
Phylogenetic reconstruction based on the concatenated nLSU + mtSSU + *tef1* sequence alignment of *Smardaea perezsilvae*. Maximum parsimony and Bayesian analyses recovered identical topologies concerning the relationships among the main clades of Pyropyxidaceae members. For each node, the following values are provided: maximum parsimony (MP ≥ 90%, left), maximum likelihood bootstrap (ML ≥ 90%, middle), and Bayesian inference posterior probability (BIPP ≥ 0.90, right). *Planamyces parisiensis* was used as the outgroup. The scale bar represents the expected number of nucleotide substitutions per site. The phylogenetic position of the new species is shown in bold.

No significant changes in tree topology, trace, or cumulative split frequencies of selected nodes were observed after approximately 3.1 million generations, so the first 2.5 million sampled trees (25%) were discarded as burn-in. To confirm that the sample size was sufficient, the parameter file was examined in Tracer v.1.7 (Rambaut et al. 2014), and it was confirmed that all parameters had an estimated sample size above 1,500. Maximum parsimony, maximum likelihood, and Bayesian inference recovered *S. perezsilvae*, supporting the existence of a new taxon distinct from related *Smardaea* species (BS = 100%, BI*p* = 1) (Fig. 2), forming a highly supported monophyletic group.

### Taxonomy

#### Marcelleina evangelinae

Taxon classificationFungiPezizalesPezizaceae

Raymundo, Sánchez-Flores, Alv.-Cortés & Esqueda sp. nov.

9D80EE8E-6E85-59BB-B33F-C4B7119FCD1F

859789

Figs 3, 4

##### Etymology.

In honor of Dr. Evangelina Pérez-Silva, a pioneer in the study of Ascomycetes from Mexico.

##### Diagnosis.

*Marcelleina evangelinae* resembles *M. persoonii* but can be recognized by wider paraphyses 3–4 µm diam. (vs. < 3 µm diam.), ascospores reticulate with a dense, complete, and united reticulum (vs. reticulate-crested with reticulum occasionally incomplete and separated), and shorter asci 120–200 × 12–13 µm (vs. 180–250 × 12–16.5 µm).

**Figure 3.**
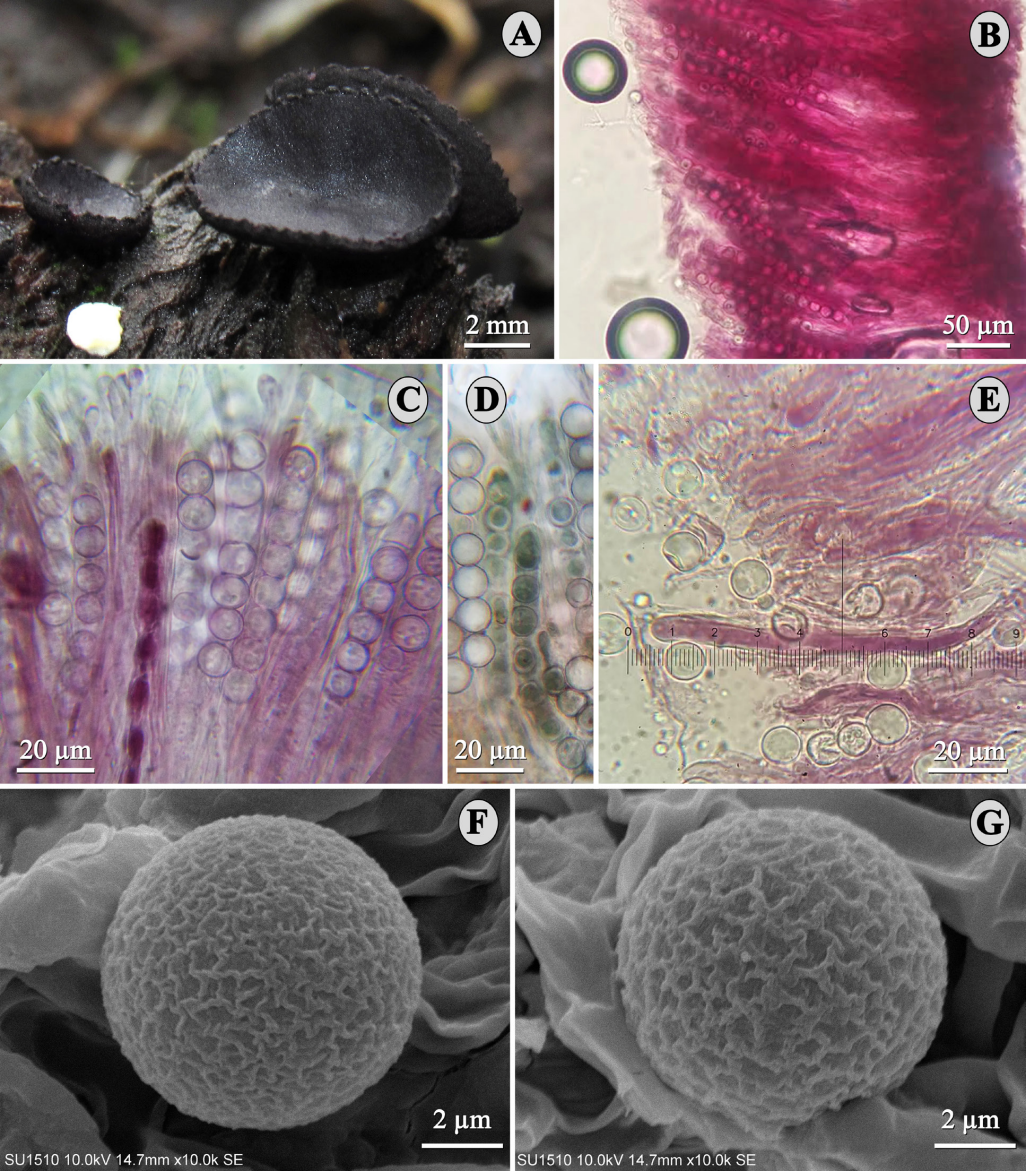
*Marcelleina evangelinae*. **A**. Apothecia; **B**. Section of the hymenium and subhymenium; **C–E**. Asci and paraphyses; **F, G**. Ascospore under SEM.

**Figure 4.**
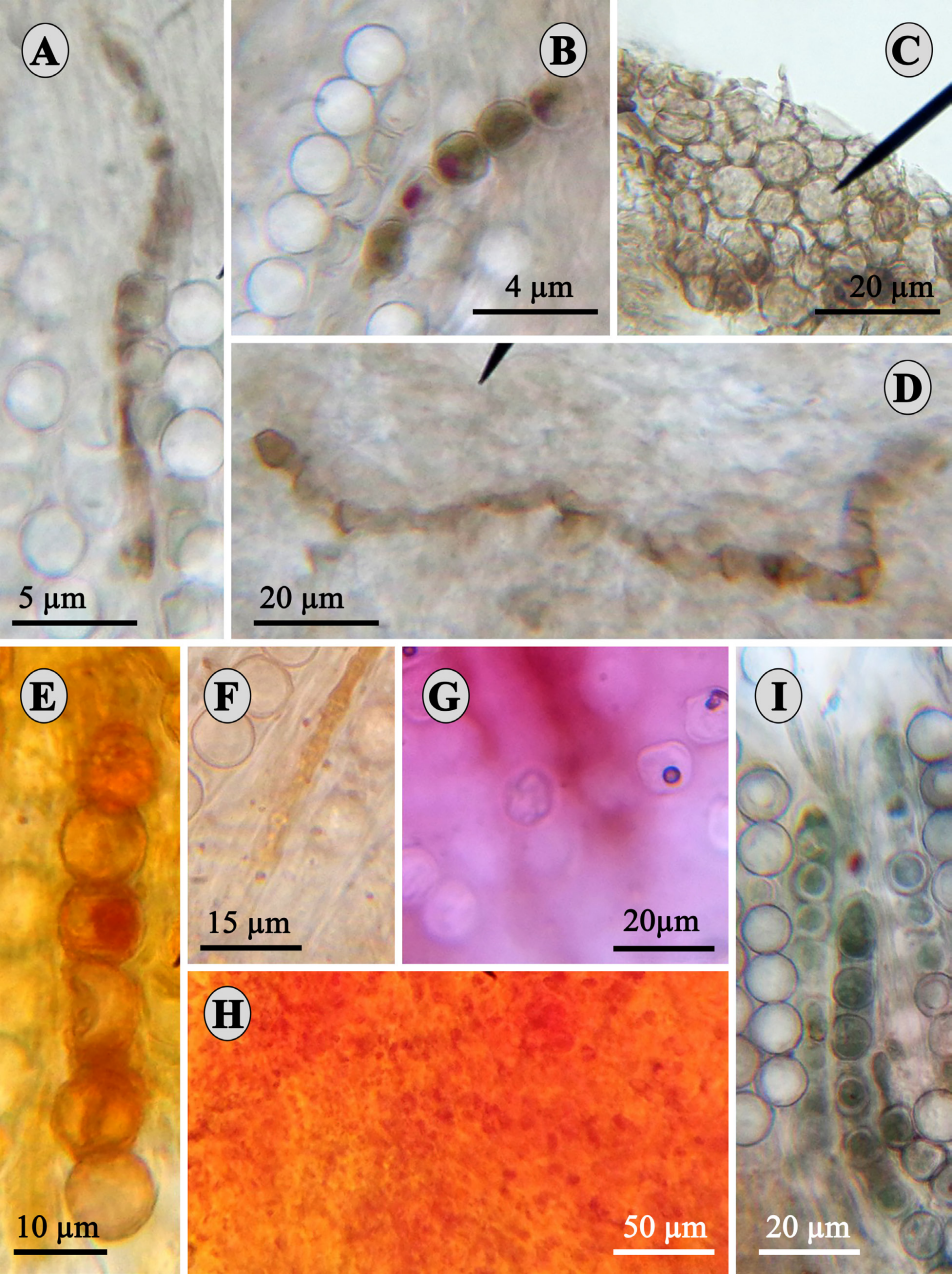
*Marcelleina evangelinae*. **A, B**. Immature asci with phenolic derivatives in ferric chloride; **C**. Ectal excipulum with phenolic derivatives; **D**. Hyphae of the medullary excipulum with phenolic derivatives; **E**. Immature ascospores with alkaloids in Dragendorff’s reagent; **F**. Paraphysis with furan derivatives in Erlich’s reagent; **G**. Paraphyses with terpenoids in sulfuric vanillin; **H**. Medullary excipulum with alkaloids; **I**. Immature ascospores with peptides in bromophenol blue.

##### Holotype.

Mexico • Sierra Madre Occidental, Sonora State, Yécora municipality, Los Pilares, km 301 carretera Hermosillo-Chihuahua, 28°23'48.9"N, 108°47'43.6"W, alt. 1,297 m asl, 18.IX.2021, A. Gutiérrez (holotype, ENCB!).

##### Description.

***Apothecia*** 5–9 mm diam., sessile on a broad base, cupulate when young, then flattened to discoid, flesh soft to soft-waxy and dark purple (3F3) to black, violaceous (3A8) KOH-extractable pigments; margin crenulate, concolorous; hymenium 600–800 µm thick, smooth, purple (3D7) to black in dry specimens; external surface dark purple (3F2) to black, slightly rugose. ***Ectal excipulum*** 120–150 μm thick at the margin and up to 200 µm thick at the base, of textura globulosa to angularis from base to margin, with globose cells 10–14 μm diam., mixed with subglobose to elongated cells 14–18 × 10–12 μm, up to 2 μm thick-walled, magenta (6D6), cherry (11D8) to deep magenta (13–14D8). ***Medullary excipulum*** 200–400 μm thick, of textura intricata, hyphae 4–8 μm diam., magenta to light purplish in KOH, septate, branched, thin-walled, intermixed with globose to subglobose cells, 10–16(–24) μm broad, up to 2 μm thick-walled, magenta to light purplish in KOH. ***Subhymenium*** 10–20 μm thick, of textura globulosa, with globose to subglobose cells, 4–5 μm diam., thin-walled, hyaline to magenta in KOH, mixed with a few short, branching, septate hyphae, thin-walled, 2–4 μm diam. ***Paraphyses*** 220–240 μm long, filiform, up to 20 μm beyond the asci, 3–4 μm diam., unbranched, septate, hyaline, pale yellow to light purple in KOH, with swollen apex, slightly curved, 4–6 μm in diam. at the top. ***Asci*** 120–200 × 12–13 μm, octospored, cylindrical, hyaline, inamyloid. ***Ascospores*** uniseriate, 9–11 μm (x̄ = 9.6 μm, *n* = 150, Q = 1.0), globose, hyaline in KOH, surface covered by a dense non-cyanophilous reticulum, ribs 0.5–0.8 μm high and × 0.4–0.6 μm wide.

##### Histochemistry.

This species contains phenolic derivatives in the extracellular matrix of the ectal excipulum, elongated tortuous hyphae of the medullary excipulum, paraphyses, and immature ascospores (Fig. 2). The paraphyses also contain furan derivatives, terpenoids, and peptides, which are also present in the asci and ascospores. Alkaloids were present in the extracellular matrix of the medullary excipulum and in the immature ascospores.

##### Other specimens examined.

Mexico • Sierra Madre Occidental, Sonora State, Yécora municipality, Los Pilares, km 301 carretera Hermosillo-Chihuahua, 28°23'48.9"N, 108°47'43.6"W, alt. 1,297 m asl, 18.IX.2021, F. Campas (UES10635). Estado de México, Malinalco municipality, San Simón El Alto, carretera federal San Simón El Alto – San Nicolás, 29.X.2023, P.M. Alvarez-Cortés 213 (ENCB).

##### Notes.

*Marcelleina evangelinae* grows gregariously on decayed wood under *Cupressus lusitanica* Mill. in temperate forests. It has only been documented in Mexico, in the states of Sonora and Estado de México. *M. evangelinae* is close to *M. persoonii*, with ascospores of 9–11 μm diam. (Sammut et al. 2023). The former has ascospores ornamented with a dense, complete, and united reticulum and asci of 120–200 × 12–13 µm, while the latter has ascospores with a complete reticulum, rarely incomplete and separated, with larger and tortuous meshes, and asci of 180–250 × 12–16.5 µm (Moravec 1987; Martínez-Gil and Caballero 2016; Olariaga et al. 2020). *M. persoonii* was described from Europe, while *M. evangelinae* was described from North America (Mexico).

### Key to *Marcelleina* species worldwide

**Table:** 

1	Ascospores globose to subglobose, smooth	**2**
–	Ascospores ornamented	**3**
2	Ascospores globose 9–12 μm, smooth; apothecia 5–9 mm diam., patelliform to flat, dark violaceous to black violaceous	***M. benkertii***
–	Ascospores globose 14–18 μm, smooth; apothecia 2–4 mm diam., cupulate to discoid, brown sanford, amber to pale brown	***M. chopraiana***
3	Ascospores reticulate; apothecia bluish violaceous, pale violaceous, or dark purple	**4**
–	Ascospores tuberculate; apothecia dark violaceous	***M. tuberculispora***
4	Apothecia purple to dark purple; paraphyses slightly curved, septate, 3–4 μm diam; ascospores 9–11 μm diam., reticulate with ridges up to 1 μm high	***M. evangelinae***
–	Apothecia bluish violaceous or pale violaceous	**5**
5	Apothecia bluish violaceous; paraphyses slightly enlarged to bifurcate < 3 μm diam.; ascospores 9–11 μm diam., reticulate-crested	***M. persoonii***
–	Apothecia pale violaceous, paraphyses curved > 3 μm diam	**6**
6	Apothecia 3–10 mm diam.; paraphyses filiform, enlarged, curved, 3–4 μm diam.; ascospores 9–11 μm diam., tuberculate with spines	***M. georgii***
–	Apothecia 3–6(–10) mm diam.; paraphyses filiform, curved, 5–6.5 μm diam.; ascospores 8–11 μm diam., reticulate	***M. rickii***

#### Smardaea perezsilvae

Taxon classificationFungiPezizalesPyropyxidaceae

Sánchez-Flores, J. García, Esqueda & Raymundo sp. nov.

3AC19A1C-DB9D-54F3-ACF4-0C5816A8DD3F

859790

Figs 5, 6

##### Etymology.

In honor of Dr. Evangelina Pérez-Silva, a pioneer in the study of Ascomycetes from Mexico.

##### Diagnosis.

*Smardaea perezsilvae* resembles *S. amethystina* but can be recognized by apothecia 5–11 mm diam., deep violet to dark violet (vs. 4–20 mm diam., dark purple to black in *S. amethystina*), paraphyses 3–4 µm diam. with a few septa (vs. 4–5 µm diam., septate), ascospores 20–26 × 10–12 µm with ridges and a few pustules (vs. 19.5–22 × 11–12.5 µm with thick and rounded pustules).

**Figure 5.**
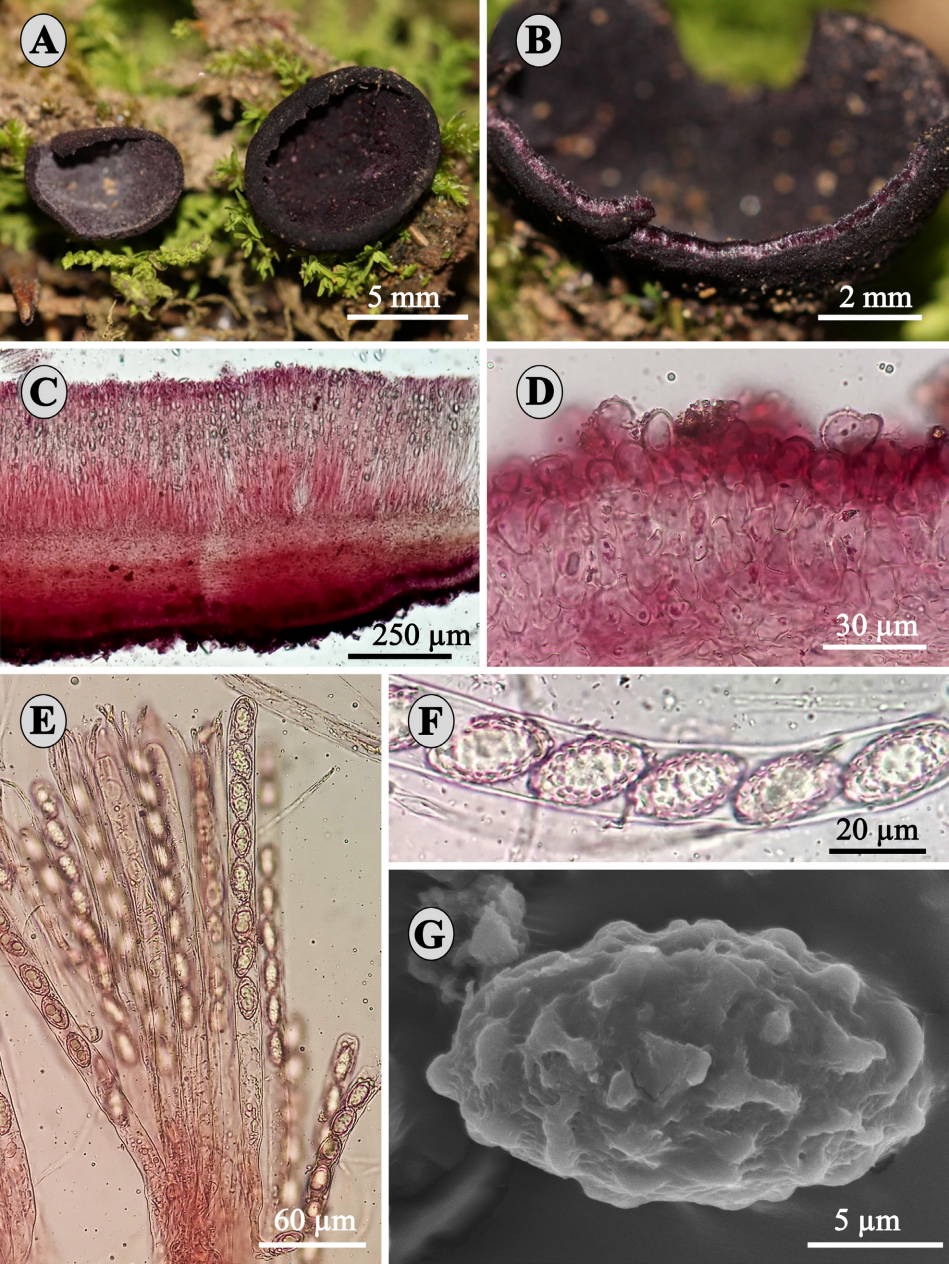
*Smardaea perezsilvae*. **A**. Apothecia; **B**. Close-up view of a sectioned apothecium in the field; **C**. Section of an apothecium; **D**. Ectal excipulum; **E**. Asci and paraphyses; **F**. Ascus and ascospores; **G**. Ascospore under SEM.

**Figure 6.**
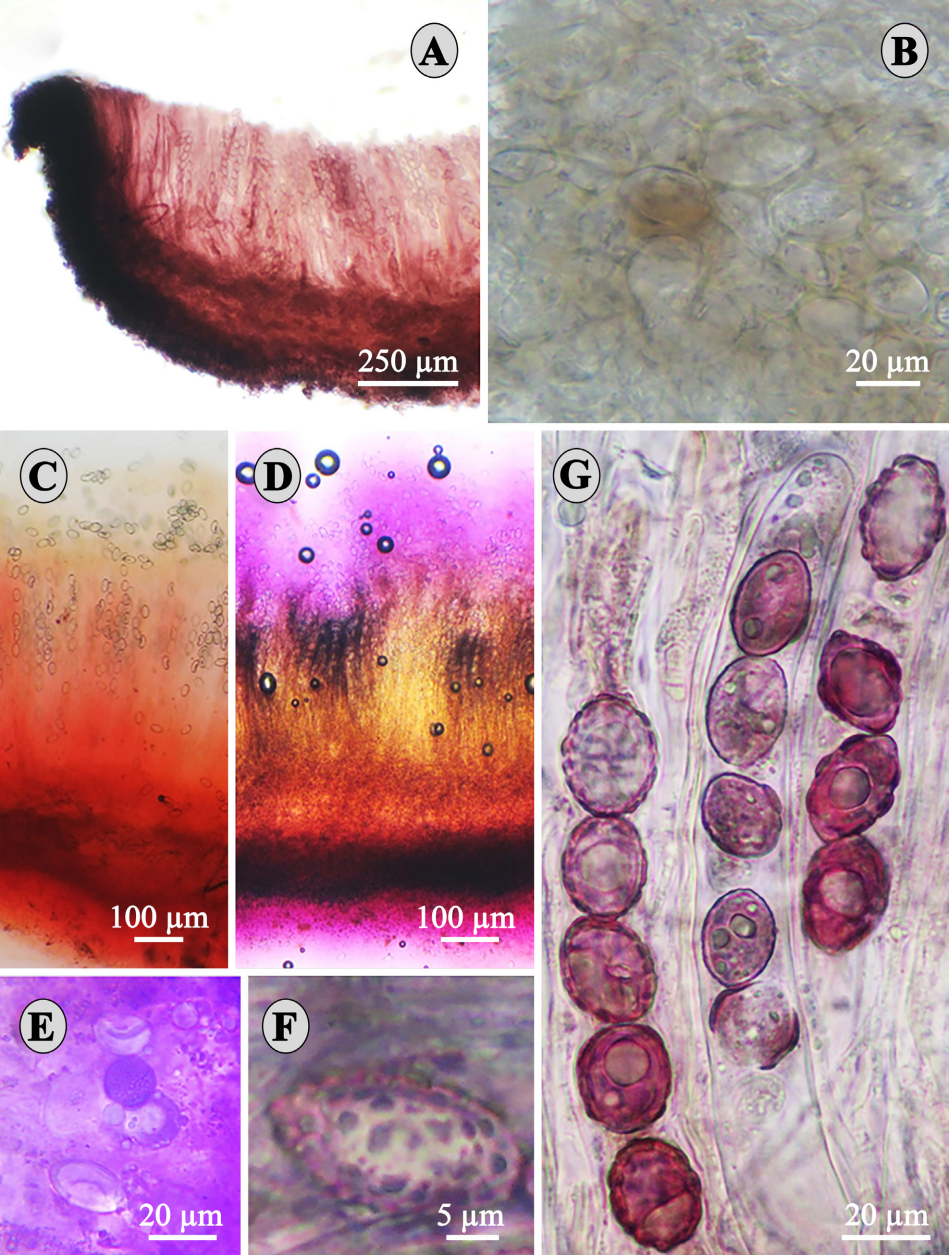
*Smardaea perezsilvae*. **A**. Section of an apothecium in water; **B**. Medullary excipulum with phenolic derivatives in ferric chloride; **C**. Hymenium with furan derivatives in Erlich’s reagent; **D**. Section of an apothecium with terpenoids in sulfuric vanillin; **E**. Detail of medullary excipulum guttules with terpenoids in sulfuric vanillin; **F, G**. Ascospore ornamentation with peptides in bromophenol blue.

##### Holotype.

Mexico • Transversal Neovolcanic Axis, Sierra Nevada, Estado de México, Texcoco municipality, Monte Tlaloc, 19°26'11.48"N, 98°45'50.73"W, alt. 3106 m asl, 18.IX.2022, M. Sánchez 2980 (holotype, ITCV!).

##### Description.

***Apothecia*** 5–11 mm diam., sessile, cupulate when young to slightly discoid when mature, hymenium smooth, deep violet (18E8) to dark violet (18F8), KOH-extractable pigments; margin entire and straight when young, irregular to crenate when mature; external surface dark violet (18F8, 18F7). ***Ectal excipulum*** 44–75 µm thick, of textura angularis to globulosa, greyish violet (18E6, 18D7) to deep violet (17D8, 17E7), cells 11–25 × 7–15 µm, up to 2 µm thick-walled. ***Medullary excipulum*** 60–114 µm thick, of textura intricata, hyphae 3–7 µm diam., deep violet (17D8, 17E7). ***Subhymenium*** 30–38 µm thick, deep violet (17D8, 17E7). ***Hymenium*** 285–315 µm thick. ***Paraphyses*** 220–240 × 3–4 µm, filiform, hyaline, unbranched, with a few septa, apex slightly swollen, containing pigments deep violet (17D8, 17E7). ***Asci*** 285–315 × 10–15 µm, octospored, cylindrical, hyaline, inamyloid. ***Ascospores*** uniseriate, 20–26 × 10–12 µm (x̄ = 23 × 10.9 µm, *n* = 150, *Q* = 2.1), ellipsoid to fusoid, hyaline, crested with a few pustules non-cyanophilous, 0.5–1.4 µm high, containing two large oil drops, often merged in rehydrated material.

##### Histochemistry.

This species contains a few phenolic derivatives in the paraphyses and the extracellular matrix of the ectal excipulum; the asci and paraphyses contain terpenoids. The medullary excipulum showed alkaloids, and the ectal excipulum showed terpenoids. The perisporium and guttules of ascospores showed peptides and terpenoids, respectively. Likewise, peptides were observed as small dots inside the asci.

##### Other specimens examined.

Mexico • Transversal Neovolcanic Axis, Sierra Nevada, Estado de México, Texcoco municipality, Monte Tlaloc, 19°26'11.48"N, 98°45'50.73"W, alt. 3106 m asl, 18.IX.2022, M. Sánchez 2956 (ITCV), 2965 (ITCV), 2967 (ITCV), 2973 (ITCV).

##### Notes.

*Smardaea perezsilvae* grows gregariously on soil under *Abies religiosa* in temperate forests and has only been reported from Mexico (Estado de México). *S. perezsilvae* is mainly distinguished from *S. amethystina* by its spore size and ornamentation. The former has slightly larger ascospores, 20–26 × 10–12 µm vs. 19.5–22 × 11–12.5 µm, with ridges and a few pustules, while the latter has ascospores with thick and rounded pustules (Iglesias 2011).

### Key to worldwide *Smardaea* species

**Table:** 

1	Ascospores globose to subglobose	**2**
–	Ascospores ellipsoid to fusoid	**6**
2	Ascospores smooth to minutely verrucose	**3**
–	Ascospores verrucose or reticulate	**4**
3	Ascospores smooth 8–10.5(11.5) μm diam.; apothecia (3)5–15 mm diam., hymenium fuscous-black, violaceous black to blackish-purple	***S. australis***
–	Ascospores smooth to minutely verrucose (8.5)9.8–10.8(11) μm; apothecia 10–20 mm diam., hymenium violaceous purple to blackish purple	***S. planchonis***
4	Ascospores 10–11 μm diam., reticulate; apothecia 10–40 mm diam., hymenium violet to purple	***S. reticulosperma***
–	Ascospores verrucose	**5**
5	Ascospores 9–12 μm diam., verrucose, warts up to 1 μm high; apothecia 5–15 mm diam., hymenium violet to purple	***S. verrucispora***
–	Ascospores 13–15 × 12–14 μm, verrucose; apothecia 4–5 mm diam., dark violaceous brownish to almost black	***S. marchica***
6	Ascospores smooth to finely verrucose	**7**
–	Ascospores coarsely verrucose to reticulate	**8**
7	Ascospores 12–14 × 8–9(10) μm, smooth; apothecia 8–15 mm diam., hymenium violet	***S. ovalispora***
–	Ascospores finely verrucose, warts angular to irregular up to 0.7 μm high, 19–26(30) × 9–12(13); apothecia 5–12 mm diam., hymenium purple to dark purple	***S. protea***
8	Ascospores finely reticulate when mature, ornamentation up to 0.5 μm high, 16–18 × 7.5–9.5 μm, apothecia 3–6 mm diam.; hymenium purple to black-purple	***S. microspora***
–	Ascospores coarsely verrucose to tuberculate	**9**
9	Apothecia 2.5–7 mm diam.; ascospores (20)22–24(25) × (10)11.5–13.6(15), coarsely warted to reticulate when mature, ornamentation 1–3 µm high	***S. purpurea***
–	Apothecia olive, purple, dark purple	**10**
10	Apothecia 3–8 mm diam., olive gray, olive brown, dull green to greyish green; ascospores 22–28 × 10–14(15) μm with rounded warts or tubercles	***S. isoldae***
–	Apothecia purple, dark purple, blackish violet	**11**
11	Apothecia 4–20 mm diam., dark purplish violet or blackish violet; ascospores 19.5–22 × 11–12.5 μm with pustules thick and rounded	***S. amethystina***
–	Apothecia 5–11 mm diam., purple to dark purple; ascospores 20–26 × 10–12 µm, crested with few pustules	***S. perezsilvae***

## Discussion

*Marcelleina* species have been described mainly from Europe, except *M. chopraiana* from India (Batra 1960), while *M. evangelinae* is the first record for Mexico in the Americas. It grows in forests, mainly under *Cupressus*, at an altitude between 1,300 and 1,750 m a.s.l., in a temperate-humid climate in Malinalco, Estado de México, and Yécora, Sonora, Mexico. This genus is commonly terrestrial, sometimes associated with calcareous soil (Sammut et al. 2023), while *M. evangelinae* grows on decayed wood, which could be a characteristic of this species. The type of *M. atroviolacea* was described from France, although records exist for the USA and Argentina (Rifai 1968); however, these American collections would correspond to *Smardaea planchonis* (Dunal ex Boud.) J. Moravec (Catcheside et al. 2017), because *M. atroviolacea* is synonymized with *S. planchonis* (Korf and Zhuang 1991). In addition, Uzun et al. (2018) reported ascospores 9–12 µm diam., while Rifai (1968) reported 8.5–10 µm diam. for Australian material, which probably corresponds to *S. australis* and not the true European *M. atroviolacea* (= *S. planchonis*). At the genus level, some species have been combined in genera such as *Iodomarcelleina*, *Scotopezia*, and *Smardaea*, among others (Sammut et al. 2023).

In the phylogeny of the *Ionopezia*–*Marcelleina* clade, the genera *Ionopezia*, *Iodomarcelleina*, and *Scotopezia* are grouped into a single family, Pezizaceae (Sammut et al. 2023). Within the basal *Marcelleina* clade, M. cf. benkertii, *M. tuberculispora*, and *M. mediterranea* cluster together (Fig. 1). The latter two species require a thorough review of their synonymy. All of them have dark purple apothecia and globose and tuberculate ascospores, growing in association with mosses in Europe (Italy, the Netherlands, and Denmark). *M. evangelinae* is the only species that grows on decaying wood, with globose and reticulate spores.

*Marcelleina benkertii*, *M. rickii*, and *M. persoonii* are phylogenetically grouped together. They have globose and reticulate ascospores, except *M. benkertii*, which has smooth ascospores. They are distributed in Spain, France, and Denmark (Moravec 1987; Lantieri and Pfister 2010; Olariaga et al. 2020). *M. evangelinae* has dark purple apothecia 5–9 mm diam. and ascospores 9–11 µm diam., reticulate with dense ridges, while *M. persoonii* has bluish violaceous apothecia 0.5–3.5 mm diam. and ascospores 9–11 µm diam., reticulate-crested, with ridges 0.3–0.6 µm high. *M. rickii* has grayish violet apothecia 3–6 mm diam. and ascospores 8–11 µm diam., with an incomplete reticulum made up of isolated ridges, 0.3–0.8(1.2) µm high, and is also close (Dennis 1981; Moravec 1987; Häffner 1995). *M. persoonii* was also recorded from the United States of America at the University of Minnesota’s Biological Station, with ascospores 7–9 µm diam. covered with warts and ridges that form an incomplete reticulum (Pfister 1985). An uncertain nLSU sequence from the USA was placed in a clade with other sequences of *M. persoonii* from Norway, Denmark, and Spain by Olariaga et al. (2020).

*Smardaea* was reported from the Americas as *Lamprospora amethystina* (Quél.) Seaver from Iowa, USA (Seaver 1914). However, Seaver’s description is insufficient for a morphological comparison with *S. perezsilvae*. *Smardaea amethystina* was first described from France with pustule-ornamented ascospores (Brummelen 1967; Svrček 1969). The mean spore size of *S. perezsilvae* is larger, and its ornamentation consists of ridges and a few pustules. Phylogenetically, *S. verrucispora* is related to *S. perezsilvae* and belongs to the same clade (Fig. 2). However, their micromorphological characteristics differ in spore size, shape, and ornamentation. Indeed, *S. verrucispora* has globose, verrucose ascospores, 9–12 µm diam. (Donadini 1976; Agnello et al. 2019). *S. amethystina* is a sister clade to *S. reticulosperma*, but it is distantly related to *S. perezsilvae*, despite their morphological similarity, differing in spore size and ornamentation. *S. amethystina* has ascospores 19.5–22 × 11–12.5 µm with pustules (Brummelen 1969; Benkert 2005; Iglesias 2011) vs. *S. perezsilvae* 20–26 × 10–12 µm, crested and with a few pustules.

In Mexico, *S. isoldae* was previously described from the montane cloud forest of Hidalgo (Raymundo and Valenzuela 2021). Both taxa differ in vegetation type, habit, and morphological characteristics. *S. isoldae* has olive-colored apothecia and ascospores with warts or rounded tubercles, while *S. perezsilvae* grows in temperate mixed forests, with purplish apothecia and ascospores crested with a few pustules. Molecular data for *S. isoldae* would be interesting because this is the only species in the genus with such an olive color. The phylogenetic analysis of relationships in *Smardaea* is difficult because only three of the 11 described species have been sequenced with nLSU and approximately five with ITS. Therefore, a phylogenetic analysis is more complicated due to the scarcity of sequences. *S. verrucispora*, *S. perezsilvae*, and *S. amethystina* grow on conifers. The first has globose ascospores, while the latter two have ellipsoidal to fusiform ascospores. *S. reticulosperma* also possesses globose ascospores but is more related to *Micronematobotrys verrucosus* Xiang, Sun & L.D. Guo, a species known only from its asexual phase, which forms *Botrytis*-type conidia and conidiophores (Fig. 2). It was isolated from leaves of *Quercus liaotungensis* Koidz. and *Ulmus macrocarpa* Hance from China, suggesting that some *Smardaea* species could be endophytes.

Purple pigments have been observed in several species of *Galactinia*, *Ionopezia*, *Marcelleina*, *Peziza*, and *Sarcosphaera*, belonging to Pezizaceae (Medel et al. 2013). They are mainly contained in paraphyses, while in *Saccobolus* and *Ascobolus* (Ascobolaceae) they are present in the episporium of ascospores, which turn reddish with KOH, a characteristic reaction of anthraquinones (Alvarez-Cortés et al. 2025). *Smardaea* and purple species of *Jafnea* have been described in Pyropyxidaceae (Zeng et al. 2022). All species of Sarcosomataceae have apothecia with blackish to black tones (Liu et al. 2025). The chemical structure of these pigments is not known with certainty. It is necessary to isolate, purify, and elucidate the molecular structure to identify the chemical groups these organisms synthesize. In Sarcosomataceae, spirobisnaphthalenes have been reported to be the urnucratines of *Urnula craterium* (Schwein.) Fr. (Liu et al. 2012). *Galiella rufa* (Schwein.) Nannf. & Korf has galiellalactones with monocyclic to tricyclic structures (Dey et al. 2023). An endophytic fungus of Sarcosomataceae sp. produces sarcosenones A–C (Hou et al. 2020), isocoumarins, and cyclohexenones (Tian et al. 2015). However, these conjugated molecules do not generate color. Furthermore, there is no chemical information on Ascobolaceae, Pezizaceae, and Pyropyxidaceae. The histochemical data for *Marcelleina evangelinae* and *Smardaea perezsilvae* indicate that their pigments contain phenolic groups.

Although the Pezizales are among the best-known groups of Ascomycota, new species continue to be described, generally found in temperate coniferous forests, which are ecologically important in their roles in ectomycorrhizal associations and organic matter decomposition. However, these ecosystems are the most susceptible and threatened by climate change, soil loss, and forest fires (Alfaro-Reyna et al. 2022). In these vulnerable ecosystems, it is necessary to intensify efforts to know and understand the diversity of Pezizales in Mexico. The studied species produce promising metabolites, including purple pigments with phenolic groups, with antioxidant and anti-inflammatory activity. A detailed analysis is required to determine the chemical structures involved.

In conclusion, the morphological, histochemical, and multi-locus phylogenetic analyses in this research revealed two new species of Pezizales from the temperate forests of Mexico. Systematic studies are recommended to clarify the phylogenetic relationships within *Marcelleina* and *Smardaea*, as well as to provide new insights into intergeneric criteria for a better-integrated classification within Pezizales.

## Supplementary Material

XML Treatment for Marcelleina evangelinae

XML Treatment for Smardaea perezsilvae

## Additional information

### Artificial Intelligence (AI) use

The authors accept full responsibility for the content of the manuscript, including the disclosure of any use of AI.

No AI tools were used in the preparation of this manuscript.

### Author ORCIDs

T. Raymundo https://orcid.org/0000-0002-7525-0034

M. Sánchez-Flores https://orcid.org/0000-0001-5630-3084

C.R. Martínez González https://orcid.org/0000-0002-0256-0840

P.M. Alvarez-Cortés https://orcid.org/0009-0009-3235-6961

J. García-Jiménez https://orcid.org/0000-0001-9290-1460

M. Esqueda https://orcid.org/0000-0003-0132-1810

### Data availability

All of the data that support the findings of this study are available in the main text.
